# Long noncoding RNA MYLK-AS1 promotes growth and invasion of hepatocellular carcinoma through the EGFR/HER2-ERK1/2 signaling pathway

**DOI:** 10.7150/ijbs.43062

**Published:** 2020-04-27

**Authors:** Juan Liu, Si-yuan Zhao, Qiwei Jiang, Yuanyuan Qu, Xiaomei Huang, Jundong Du, Wanjun Sun, Qinong Ye

**Affiliations:** 1 Department of Medical Molecular Biology, Beijing Institute of Biotechnology, Beijing 100850, China.; 2 Department of Hematology, PLA Rocket Force Characteristic Medical Center, Beijing 100088, China.; 3 Medical unit, 91638 Troops, PLA, Beijing 102202, China.; 4 Department of Surgery, Hebei Yanda Hospital, Hebei 065201, China.

**Keywords:** LncRNA, MYLK-AS1, hepatocellular carcinoma, EGFR, HER2

## Abstract

The epidermal growth factor receptor (EGFR) family members EGFR and HER2 play pivotal roles in oncogenesis and tumor progression. Anticancer drugs targeting EGFR and HER2 have been developed. Long noncoding RNAs (lncRNAs) have been reported to regulate cancer development and progression through signaling pathways. However, lncRNAs that regulate EGFR and HER2 expression remain unknown. Here, we show that lncRNA myosin light chain kinase-antisense RNA 1 (MYLK-AS1) promotes EGFR and HER2 expression and activates their downstream signaling pathway. MYLK-AS1 increases hepatocellular carcinoma (HCC) cell proliferation, migration, and invasion *in vitro*. Consistently, MYLK-AS1 knockdown hinders tumor growth *in vivo*. Mechanistically, MYLK-AS1 enhances HCC cell proliferation, migration, and invasion through stimulating the EGFR/HER2-extracellular signal-regulated kinase 1/2 (ERK1/2) signaling pathway. In addition, MYLK-AS1 is overexpressed in HCC patients and negatively correlated with HCC prognosis. Thus, MYLK-AS1 is an upstream regulator of EGFR/HER2, and acts as an oncogene, suggesting an additional target for cancer therapeutics.

## Introduction

The erythroblastic leukemia viral oncogene homolog (ErbB) family consists of the epidermal growth factor receptor (EGFR, also known as ErbB1), ErbB2 (also known as HER2), ErbB3 (HER3) and ErbB4 (HER4), all of which are structurally related receptor tyrosine kinases (RTKs) 1. These proteins, especially EGFR/HER2, are usually activated in many malignant tumors, including hepatocellular carcinoma (HCC) 2,3. Overexpression or activation of EGFR/HER2 or their downstream signaling pathways is closely related to drug resistance, which finally leads to poor outcomes of patients 4. The main downstream signaling pathway of EGFR/HER2 includes the rat sarcoma (RAS)-RAF proto-oncogene serine/threonine protein kinase-mitogen-activated protein kinase kinase 1/2 (MEK1/2)-extracellular signal regulated kinase 1/2 (ERK1/2) pathway controlling cancer development and progression 5-7. Several approaches, including the development of antibodies and small molecule inhibitors, have been employed to target EGFR and HER2 receptors or EGFR/HER2-modulated effects for cancer therapy 8-11. Although clinical studies reveal promising results when using antibodies or inhibiters of EGFR/HER2 or both approaches, many patients are resistant to these treatments by activating or overexpressing alternative pathways and present a more aggressive phenotype, with more migratory and invasive cells compared to the non-resistant tumors. Therefore, elucidating molecular mechanisms of regulating EGFR/HER2 and their downstream signaling pathways are urgently needed.

Long non-coding RNAs (LncRNAs) with a length longer than 200 nucleotides are a class of non-protein coding transcripts 12. More and more studies indicate that lncRNAs play essential roles in oncogenesis and progression of HCC by acting on proteins, stabilizing mRNA, regulating miRNA, or signaling pathways 13-15. HCC is a large proportion of tumors worldwide and China alone accounts for about 50% of the total liver cancer rates 16. Despite great improvements in exploring molecular mechanisms of HCC progression and therapeutic treatments, the overall survival (OS) and recurrence-free survival (RFS) rates, and the 5-year survival rates are still comparatively low, which means that the underlying molecular mechanisms need to be deeply and comprehensively studied 17,18. MYLK-AS1, locating at chromosome 3, is a kind of long non coding RNA with a length of 814 bp. MYLK-AS1 was originally reported in the National Institutes of Health Mammalian Gene Collection (MGC) Program to identify putative alternative promoters of human genes but with no detailed studies 19,20. Very recently, the lncRNA expression-based risk score system for OS of HCC patients has been constructed based on six lncRNAs including MYLK-AS1 21. MYLK-AS1 was reported to be downregulated in 9 colon adenocarcinoma samples compared to paired adjacent normal tissues 22. However, the biological function of MYLK-AS1 is unknown, and the clinical significance of MYLK-AS1 still requires to be further determined.

In the present study, we used gene set enrichment analysis (GSEA) to investigate lncRNAs involved in the EGFR/HER2-ERK1/2 signaling pathway, and identified MYLK-AS1 as an upstream regulatory factor of EGFR/HER2. In HCC patients, MYLK-AS1 is up-regulated, and negatively correlated with prognosis. MYLK-AS1 promotes growth and invasion of HCC through the EGFR/HER2-RAS-RAF-MEK1/2-ERK1/2 signaling pathway.

## Materials and methods

### Data source

The RNA-seq data and clinical information of HCC were downloaded from the TCGA data portal (https://tcga-data.nci.nih.gov/) with a total of 377 samples (377 tumor samples and 41 normal tissue samples adjacent to tumors). Of these, 369 patients had complete prognostic information. Sequence data were generated using the Illumina HiSeq 2000 RNA Sequencing platform. The data were downloaded in September, 2019.

### Functional enrichment analysis

Based on lncRNAs and gene relationships calculated by the Pearson correlation coefficients, functional enrichment analysis for lncRNAs in the lncRNA signature set was performed using DAVID. Gene set enrichment analysis (GSEA) was conducted using GSEA 4.0.1 version. GSEA 4.0.1 was performed by the JAVA program (http://software.broadinstitute.org/gsea/downloads.jsp) using the MSigDB C2 canonical pathways gene set collection, which contains 1320 gene sets. Gene sets with a false discovery rate (FDR) value less than 0.05 after performing 1000 permutations were considered to be significantly enriched 23. If most sequenced genes correlated with higher expressions of lncRNAs are enriched at the top of K-RAS signaling up gene set, we speculate that these lncRNAs are positively correlated with K-RAS signaling up.

### Plasmids, siRNA, shRNA, and lentiviruses

The eukaryotic expression vectors with full length MYLK-AS1 were generated by inserting PCR-amplified fragments into pcDNA3.0 (Invitrogen) to overexpress MYLK-AS1. To knock down the expression of MYLK-AS1, ERK and EGFR, small interfering RNAs (siRNAs) were designed and synthesized by RiboBio. The sequences of siRNAs for MYLK-AS1 are 5′-CCTCTTAAATGCTATACTA-3′, 5′-GTGCGGGATTGCTGATGAA-3′, 5′-ACATTTCTGTAAAGATGAA-3′, 5′-GTTCAAGTGCATCTTCCAGC-3′, 5′-GAGAGACAAGAGCAGGACAG and CAGCCAGTGAGATTGGAAGC-3′. The sequences of siRNAs for ERK1/2 are 5′-GACCGGAUGUUAACCUUUA-3′ (ERK1); and 5′-CCAGGAUACAGAUCUUAAA-3′ (ERK2) 24. The sequence of siRNA for EGFR is 5′-GAGGAAAUAUGUACUACGAdTdT-3′ 25. According to the MYLK-AS1 siRNAs, a short hairpin RNA (shRNA) sequence aiming for MYLK-AS1 was subcloned into pSIH-H1-Puro (System Biosciences). Lentiviruses were produced by co-transfection of HEK293T cells with recombinant lentivirus vectors and pPACK Packaging Plasmid Mix (System Biosciences) using Megatran reagent (Origene), and were used to infect target cells according to the manufacturers' instructions.

### Cell lines and drug treatments

The human hepatoma cell lines, HepG_2_, BEL-7402, SMMC-7721, MHCC97-H, the human normal cell lines LO2 and HEK293T were purchased from American Type Culture Collection (ATCC), and their mycoplasma contaminations have previously been tested. Cells were usually cultured in DMEM containing 25 mM glucose (Invitrogen) and 10% fetal bovine serum (FBS) (Hyclone) in an atmosphere of 5% CO_2_ at 37°C. Five micrograms of plasmids and 50-200 nM siRNAs as indicated were transfected into cells using Lipofectamine 2000 reagent and Lipofectamine RNAiMAX, respectively, according to the manufacturers' instructions (Invitrogen and RiboBio). Cells with 70-90% confluency were suitable for plasmid transfection. Plasmids and Lipofectamine 2000 reagent were diluted in DMEM. The diluted plasmids were mixed with the diluted Lipofectamine 2000. The mixtures were incubated for 5 min at room temperature and added to cells in each dish. The transfected cells were collected after 24 h, and the collected cells were used for further study (cell proliferation, migration and invasion). For siRNA transfection, siRNA and Lipofectamine RNAiMAX reagent were diluted in DMEM without serum, and the dilutes were mixed gently. The mixtures were incubated for 10-20 min at room temperature and then added to cells of each dish. Cells were harvested after 48 h, and the harvested cells were used for further study (cell proliferation, migration and invasion). For the EGFR/HER2-ERK1/2 signaling pathway inhibition study, PD98059 (Sigma-Aldrich) and GW583340 (Sigma-Aldrich) were dissolved in DMSO. Cells were transfected with empty vector or pcDNA3.0-MYLK-AS1 plasmids. Twenty-four hours later, DMSO was added to cells of one group as control, and PD98059 (a final concentration of 25 nM) and GW583340 (a final concentration of 100 nM) were added to cells in other two groups, respectively. Cells were then used to perform proliferation, invasion and western blot assays.

### RNA isolation and reverse transcription-quantitative PCR

Total RNA was isolated using TRIzol reagent according to the manufacturer's instructions (Invitrogen). Total RNA was reverse transcribed into first strand cDNA with oligo (dT) primers using Moloney murine leukemia virus reverse transcriptase (Promega). Then, the first strand cDNA synthesis reaction mixture was used for PCR amplification in a total volume of 50 μl. qPCR was performed in triplicates in a 20 μl of reaction mixture containing 10 μl of SYBR Premix Ex Taq Master Mix (2×) (Takara), 0.5 μM of each of the primers and 10 ng cDNA. The relative expression was calculated by the comparative Ct method. Results were normalized to the expression of β-actin. Specific primer sequences were listed as follows: MYLK-AS1 forward, 5'-TTGCAGTGTTCAGCACTGGCAC-3', reverse, 5'-ATTCGACGACCAGTGTTTCAGT-3'; β-actin forward, 5′-ATCACCATTGGCAATGAGCG-3′, reverse, 5′-TTGAAGGTAGTTTCGTGGAT-3′.

### Cell proliferation, migration and invasion assays

For cell proliferation assay, the transfected cells were replated into 96-well plates at 3000 cells/well. Cell proliferation was determined by the CCK-8 Kit (Dojindo Laboratories) according to the manufacturer's instructions. Briefly, ten microliters of CCK-8 solution were added to cultured cells in each well, followed by incubation at 37°C for 1 h. The OD values were measured at 450 nm using a microplate reader. CCK-8 assay was performed after cell attachment for the first time (Day 0) and was continuously performed every 24h as indicated. For colony formation assay, three thousand cells were seeded per well. Two weeks later, colonies were fixed with methanol and stained with 0.1% crystal violet (Sigma-Aldrich) and visible colonies were manually counted. Cell migration was determined using wound healing assay. Cells were seeded at a density of 4 × 10^4^ cells/cm^2^. On day 3, a straight scratch was made with a 200 mL pipette tip and images of the wound were acquired under the microscope with the original magnification of 100×. After 0 and 16 h, cells were photographed under the microscope and the remaining scratch area was calculated. Cell invasion was determined using Transwell chambers with 8 μm pore size according to the manufacturer's instructions (Millipore). To the top chamber, five thousand cells in serum-free medium were added. In the lower chamber, DMEM with 10% FBS was added. After 16 h, cells that had invaded through the membrane were fixed with methanol, stained with crystal violet, and counted.

### Western blot analysis

Cells and tissue samples were collected and incubated in the RIPA buffer (Sigma-Aldrich) to obtain proteins. Approximately 30 μg of total proteins were loaded into each lane of 10-15% SDS-PAGE, separated in electrophoresis at a constant pressure of 160 V for about 1-2 h, and transferred onto nitrocellulose membranes (Millipore). After blocking, the membranes were then incubated with primary antibodies against EGFR (Santa Cruz Biotechnology), phosph-EGFR (Santa Cruz Biotechnology), HER2 (Santa Cruz Biotechnology), RAS (Santa Cruz Biotechnology), RAF1 (Santa Cruz Biotechnology), MEK1/2 (Cell Signaling), phosph-MEK1/2 (Cell Signaling), ERK1/2 (Cell Signaling), phosph-ERK1/2 (Cell Signaling), and β-actin (Santa Cruz Biotechnology). β-actin was loaded as an internal reference. Bands were then treated with horseradish peroxidase (HRP)-conjugated secondary antibody (Santa Cruz Biotechnology). Bands were developed using chemiluminescence substance (Thermo Scientific). All experiments were conducted three times independently with similar results. A representative result for Western blots was provided.

### *In vivo* experiments

The animal experiments were reviewed and approved by the Institutional Animal Care and Use Committee of the Beijing Institute of Biotechnology, Beijing, China. The nude mice (thymus deficiency/T-cell deficiency) aged 4-6 weeks were purchased from Si-Bei-Fu Biotechnology Corporation, Beijing, China. In the subcutaneous transplantation model, mice were seeded with MYLK-AS1 shRNA-expressing MHCC97-H cells or control shRNA-expressing MHCC97-H cells (5 × 10^6^ cells suspended in 200 μL of phosphate buffered saline [PBS]) in the abdominal flank. After three weeks, the mice were harvested and the tumors were collected. The tumor weights were measured by using a precision balance. The tumor length or tumor width was measured by using a Vernier Caliper. The tumor volumes were calculated as tumor length × tumor width × tumor width/2 26,27.

### Statistical analysis

Statistical significance in the preclinical experiments was assessed by two-tailed Student's *t*-test. The Kaplan-Meier method was used to evaluate overall survival of HCC patients and the log-rank test was performed to determine the differences between survival curves. Prism 7.0 (GraphPad Software) was used to analyzed all data. The statistical significance was defined as p < 0.05 in all assays.

## Results

### MYLK-AS1 is overexpressed in HCC patients and negatively correlated with HCC prognosis

**Figure 1:**
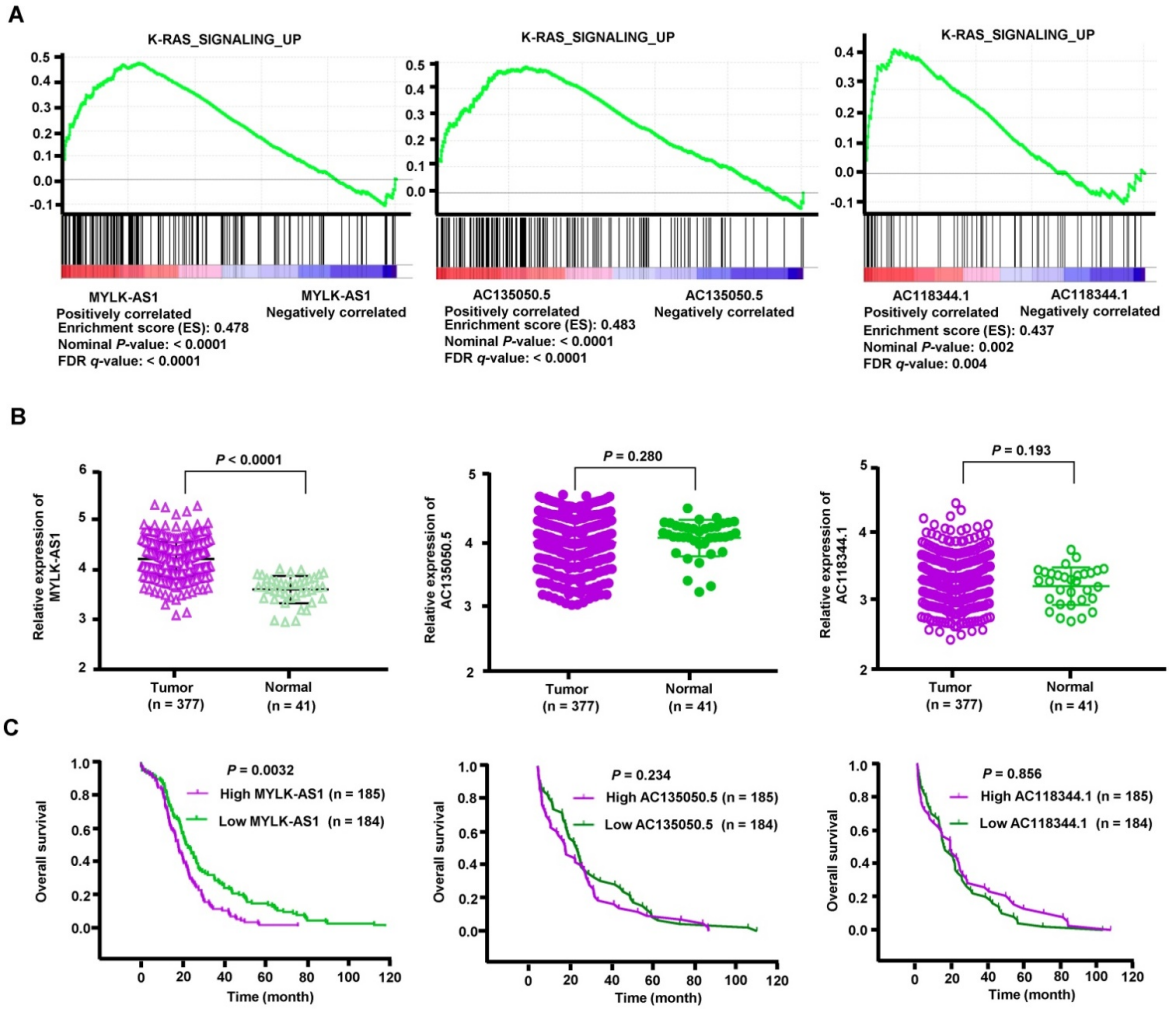
** Analysis of HCC RNA-seq data from TCGA.** (A) Cluster analysis of lncRNAs correlated with the activation of K-RAS signaling. (B) The expression levels of three lncRNAs (MYLK-AS1, AC135050.5 and AC118344.1) were compared between HCC tumor tissues and adjacent normal tissues using RNA-seq data downloaded from TCGA. (C) The relationships between the 3 lncRNAs and the HCC survival were analyzed based on clinical information of TCGA data.

To explore potential lncRNAs associated with the EGFR-RAS-ERK signaling pathway, we screened lncRNAs associated with EGFR in HCC using TANRIC (https://ibl.mdanderson.org/tanric/_design/basic/main.html). Firstly, we obtained 293 lncRNAs, out of which 131 lncRNAs were positively correlated with EGFR expression. According to the value of correlation coefficient, the top 10 lncRNAs were selected for GSEA analysis. Gene sets were considered significantly enriched if the nominal p-value was less than 0.005 and the FDR less than 0.05 based on a canonical pathways gene set from the MSigDB database 28. We identified 3 lncRNAs (MYLK-AS1, AC135050.5 and AC118344.1) correlated with the activation of K-RAS signaling, which is a critical downstream signaling pathway of EGFR/HER2 (Figure 1A). We then compared expression levels of the 3 lncRNAs between HCC tumor tissues and adjacent normal tissues using RNA-seq data downloaded from the Cancer Genome Atlas (TCGA). Compared with corresponding normal liver tissues, HCC tumor tissues exhibited significantly upregulated expression of MYLK-AS1, but not AC135050.5 and AC118344.1 (Figure 1B). Furthermore, based on clinical information of TCGA data, we stratified HCC patients into two groups by the medians of lncRNAs expression levels. The survival analysis showed that higher expression of MYLK-AS1 was significantly correlated with poorer prognosis in HCC patients (Figure 1C). Again, the expression levels of AC135050.5 and AC118344.1 had no relations with HCC prognosis (Figure 1C). Moreover, MYLK-AS1 was not associated with K-RAS signaling activation in normal samples, which is obviously different from the GSEA result in HCC tumors (Figure S1). These data indicate that MYLK-AS1 may be involved in the activation of EGFR/HER2-ERK1/2 signaling pathway in HCC.

### MYLK-AS1 promotes HCC cell proliferation* in vitro*

**Figure 2:**
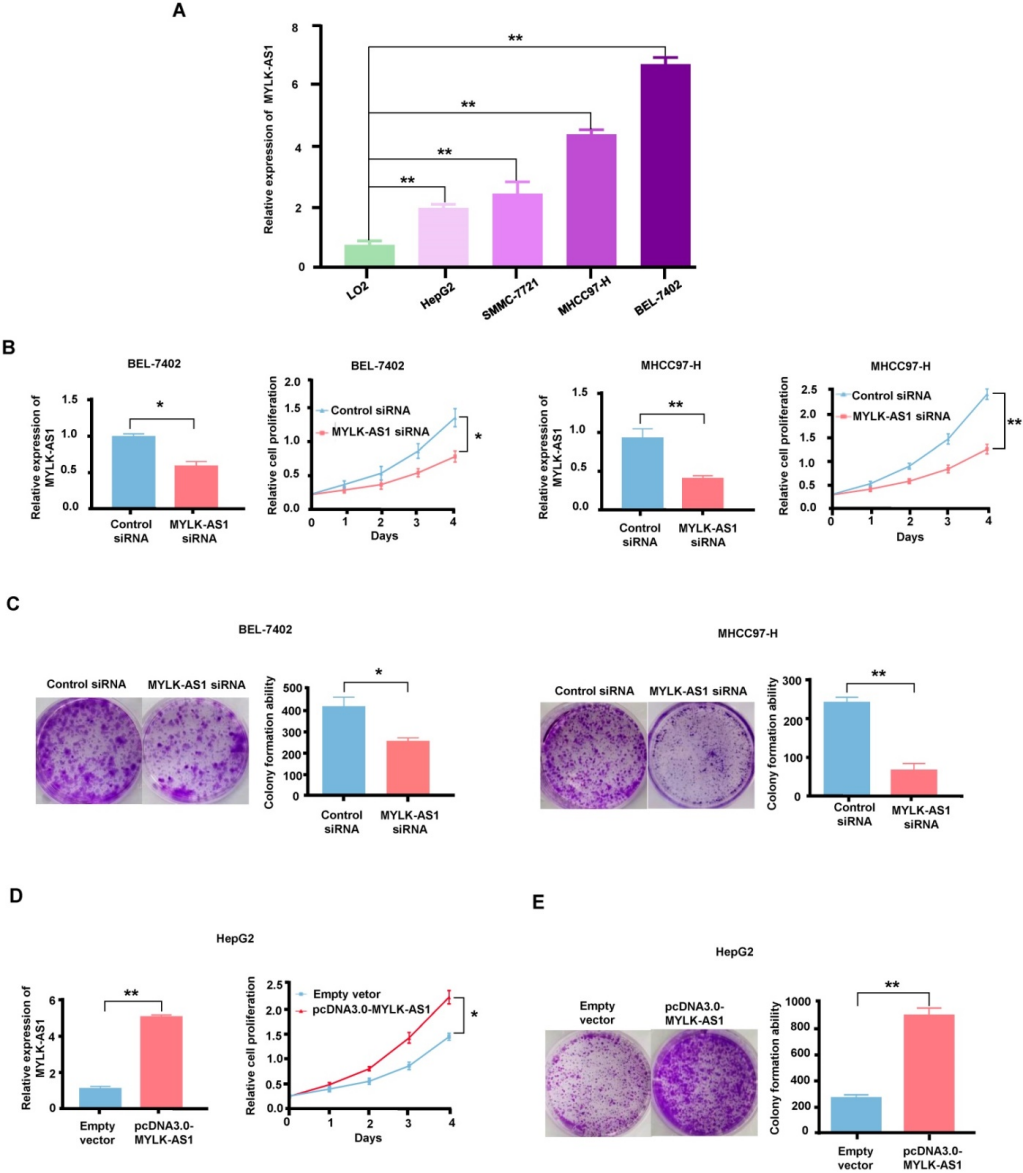
** MYLK-AS1 increases HCC cell proliferation *in vitro*.** (A) MYLK-AS1 expression was analyzed by RT-qPCR in normal hepatocyte cell line LO2 and HCC cell lines (HepG2, SMMC-7721, MHCC97-H and BEL-7402). (B and C) BEL-7402 and MHCC97-H cells were transfected with MYLK-AS1 siRNAs to knockdown the expression of MYLK-AS1. The knockdown effects were determined by RT-qPCR. Cell proliferation was measured by CCK-8 assay and colony formation assay. (D and E) HepG2 cells were transfected with pcDNA3.0-MYLK-AS1 to overexpress MYLK-AS1. The overexpression effects were determined by RT-qPCR. Cell proliferation was detected by CCK-8 assay and colony formation assay. All values shown are mean ± SD of triplicate measurements and have been repeated 3 times with similar results (*P < 0.05, ** P < 0.01).

To select HCC cell lines suitable for MYLK-AS1 overexpression and knockdown experiments, we used RT-qPCR analysis to examine the expression of MYLK-AS1 in four different HCC cell lines (HepG2, SMMC-7721, MHCC97-H, and BEL-7402) and one normal human hepatocyte cell line (LO2). MHCC97-H, and BEL-7402 cells expressed higher level of MYLK-AS1 than HepG2, SMMC-7721 and LO2, with LO2 expressing the lowest level of MYLK-AS1 (Figure 2A). Thus, to study the role of MYLK-AS1 in HCC, we chose MHCC97-H and BEL-7402 to knockdown MYLK-AS1 and HepG2 to overexpress MYLK-AS1. BEL-7402 and MHCC97-H cells transfected with MYLK-AS1 siRNAs grew more slowly than those transfected with control siRNA (Figure 2B), indicating that MYLK-AS1 knockdown in BEL-7402 and MHCC97-H cells reduces cell proliferation. Moreover, colony formation assays showed that knockdown of MYLK-AS1 in BEL-7402 and MHCC97-H cells reduced the colony number (Figure 2C). In contrast, HepG2 cells with overexpression of MYLK-AS1 proliferated significantly faster than those transfected with empty vector (Figure 2D). Consistently, the colony formation ability of HepG2 cells was remarkably increased by overexpression of MYLK-AS1 (Figure 2E). These results demonstrate that MYLK-AS1 promotes HCC cell proliferation.

### MYLK-AS1 accelerates migration and invasion of HCC cells* in vitro*

**Figure 3:**
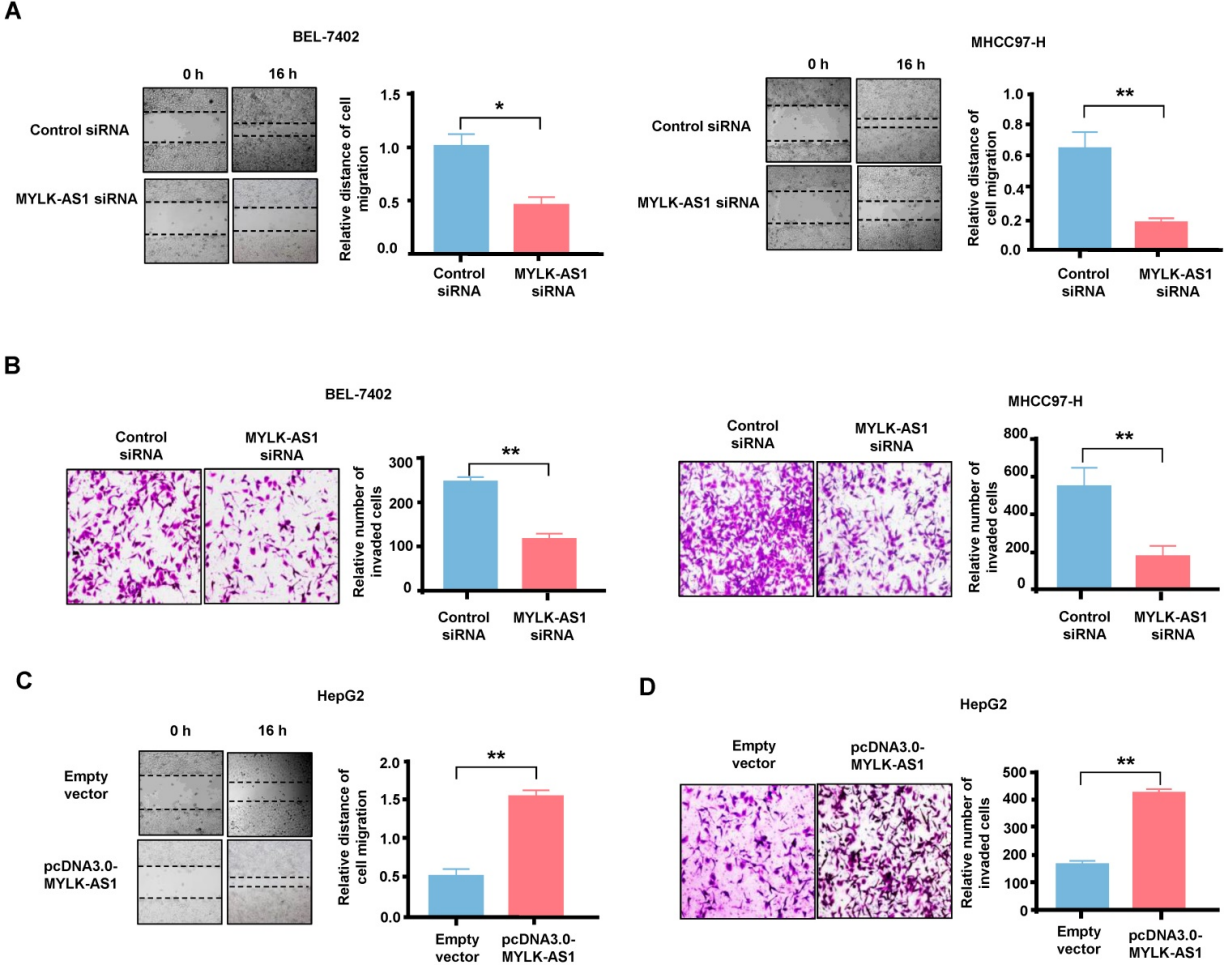
** The effects of MYLK-AS1 on HCC cell migration and invasion *in vitro*.** (A) BEL-7402 and MHCC97-H cells were transfected with MYLK-AS1 siRNAs to knockdown the expression of MYLK-AS1. Wound healing assay was performed to evaluate cell migration changes between control and MYLK-AS1 knockdown groups in BEL-7402 and MHCC97-H cells. (B) Cells were transfected as in (A). Transwell assay was performed to investigate cell invasion differences between control and MYLK-AS1 knockdown groups in BEL-7402 and MHCC97-H cells. (C and D) HepG2 cells were transfected with pcDNA3.0-MYLK-AS1 to overexpress MYLK-AS1. Wound healing assay and transwell assay were respectively used to detect cell migration (C) and invasion (D) differences between control and MYLK-AS1 overexpression groups in HepG2 cells. Data shown are mean ± SD of 3 independent experiments. (*P < 0.05 versus empty vector or control siRNA, ** P < 0.01 versus empty vector or control siRNA).

Wound-healing assay and transwell assay were performed respectively to study the biological roles of MYLK-AS1 in the migration and invasion of HCC cells. In wound healing assay, the migration ability of BEL-7402 cells with MYLK-AS1 knockdown was significantly decreased compared to that of control cells (Figure 3A). Similar results were obtained in MHCC97-H cells. Transwell invasion assay showed that MYLK-AS1 knockdown in BEL-7402 and MHCC97-H cells decreased the number of invaded cells (Figure 3B). On the contrary, the abilities of migration and invasion of HepG2 cells were markedly promoted by overexpressing MYLK-AS1 (Figure 3C and 3D). Conclusively, our data indicate that MYLK-AS1 can facilitate migration and invasion of HCC cells.

### MYLK-AS1 activates EGFR/HER2-ERK1/2 signaling pathway in HCC

**Figure 4:**
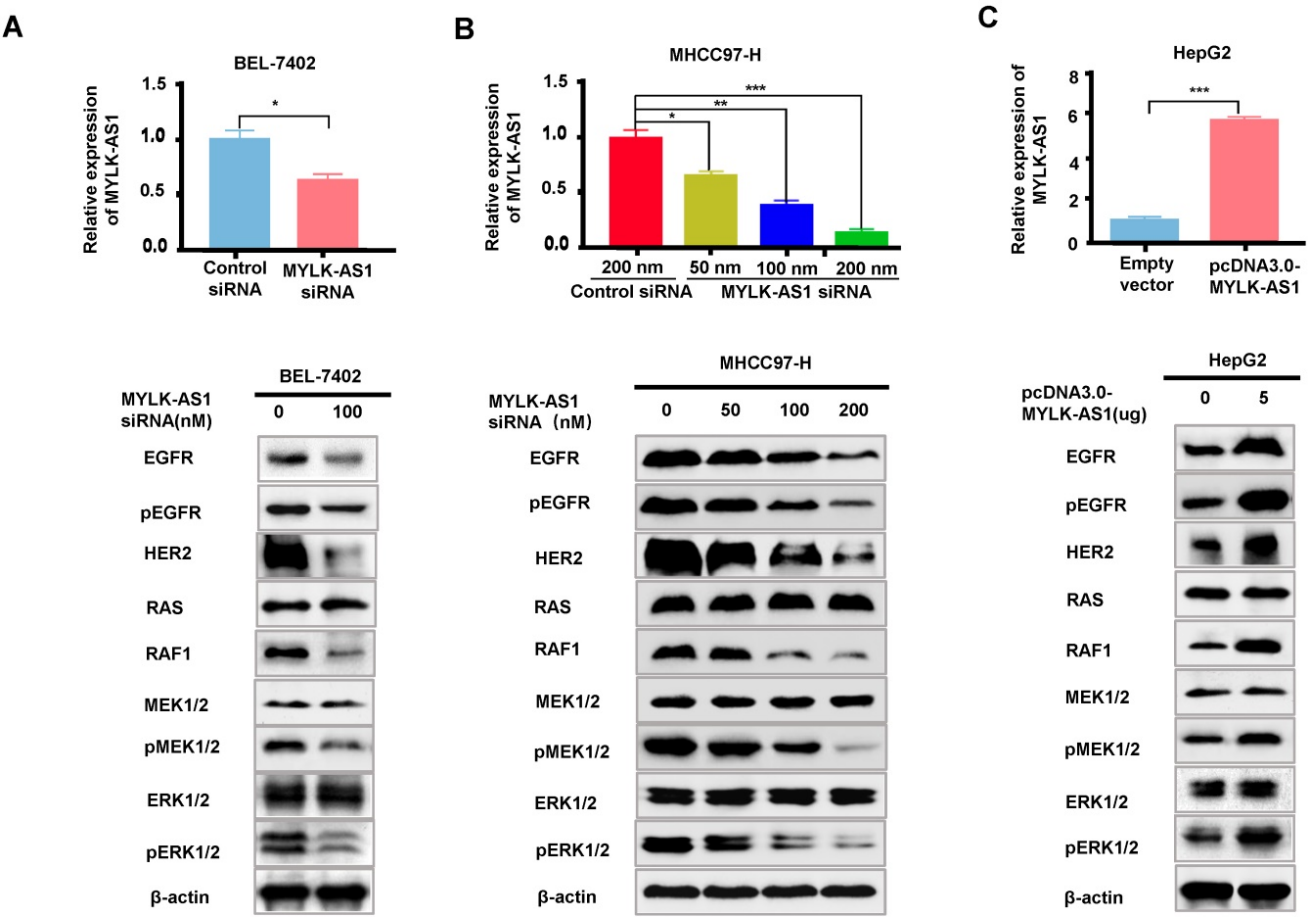
** MYLK-AS1 activates EGFR/HER2-ERK signaling pathway in HCC.** (A) BEL-7402 cells were transfected with MYLK-AS1 siRNAs (100 nM) or control siRNA (100 nM). The MYLK-AS1 knockdown effect was detected by RT-qPCR. Western blot was performed to determine the expression of EGFR/HER2-ERK signaling pathway-related genes as indicated. β-actin was used as a loading control. (B) MYLK-AS1 siRNAs (50 nM, 100 nM and 200 nM) or control siRNA (200 nM) were transfected into MHCC97-H cells. The MYLK-AS1 overexpression effect was measured by RT-qPCR. Western blot was performed as in (A). (C) HepG2 cells were transfected with MYLK-AS1 (5 μg) or empty vector. The MYLK-AS1 overexpression effect was measured by RT-qPCR. Western blot was performed as in (A). All experiments were conducted three times independently and representative immunoblot results were shown. Data were presented as the mean ± SD (*P < 0.05, **P < 0.01).

The EGFR/HER2-RAS-RAF-MEK-ERK1/2 signaling pathway plays a key role in cancer development and progression. Since MYLK-AS1 correlates with the activation of K-RAS signaling, we investigated whether MYLK-AS1 modulates expression of EGFR and HER2, the K-RAS upstream regulators, as well as RAF1, MEK1/2 and ERK1/2, the K-RAS downstream targets. MYLK-AS1 knockdown in BEL-7402 and MHCC97-H cells decreased protein expression of EGFR, pEGFR, HER2 and RAF1, but not K-RAS, MEK1/2 and ERK1/2 (Figure 4A and 4B). Although MYLK-AS1 knockdown did not alter MEK1/2 and ERK1/2 expression, knockdown of MYLK-AS1 reduced phosphorylation of MEK1/2 and ERK1/2, indicating that MYLK-AS1 knockdown inhibits activation of MEK1/2 and ERK1/2. Moreover, a dose dependent effect was observed when increasing amounts of MYLK siRNA were transfected into MHCC97-H cells (Figure 4B). In contrast, MYLK-AS1 overexpression in HepG2 cells increased EGFR, pEGFR, HER2 and RAF1 expression as well as phosphorylation of MEK1/2 and ERK1/2 (Figure 4C). These data suggest that MYLK-AS1 is an upstream regulatory factor of EGFR/HER2 and stimulates EGFR/HER2-ERK signaling pathway in HCC.

### MYLK-AS1 regulates proliferation and invasion of HCC cells through the EGFR/HER2-ERK1/2 signaling pathway

**Figure 5:**
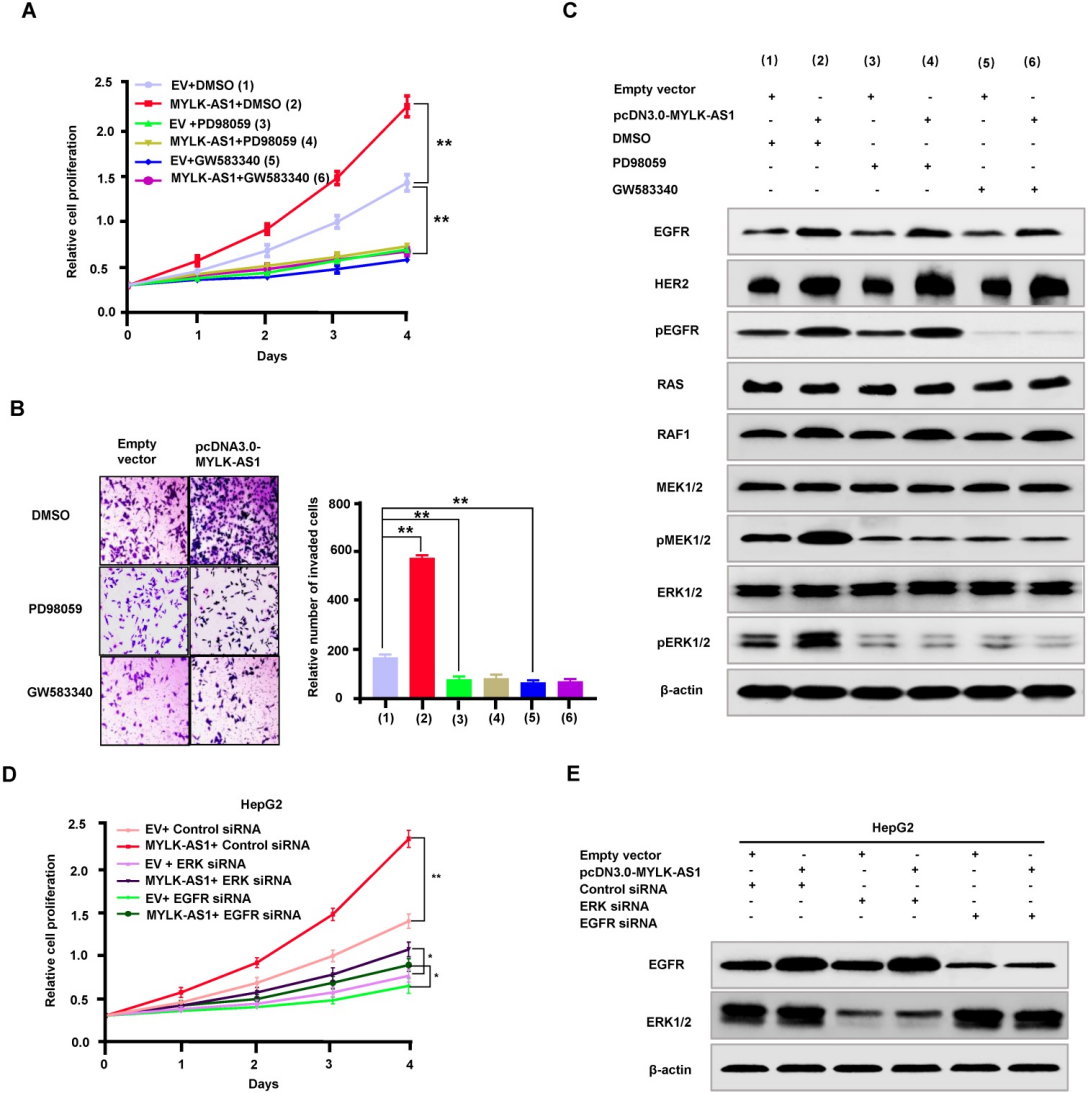
** MYLK-AS1 regulates proliferation and invasion of HCC cells through the EGFR/HER2-ERK signaling pathway.** (A) HepG2 cells were transfected with pcDNA3.0-MYLK-AS1 (MYLK-AS1) vector or empty vector (EV). Cells were treated with PD98059 or GW583340 as indicated, with DMSO as control. Cell proliferation was then determined by CCK-8 assay. All values shown are mean ± SD of triplicate measurements and have been repeated 3 times with similar results (** P < 0.01 at day 4). (B) HepG2 cells were transfected as in (A). The cell invasion changes were detected by transwell assay. All experiments were conducted three times independently, and the data were presented as the mean ± SD (**P < 0.01). (C) Representative immunoblot of HepG2 cells transfected as in (A) with the indicated antibodies. The experiments have been repeated 3 times with similar results. (D and E) HepG2 cells were transfected with ERK siRNA or EGFR siRNA as indicated, with control siRNA as control. Twenty four hours later, the cells were transfected with pcDNA3.0-MYLK-AS1 (MYLK-AS1) vector or empty vector (EV) as indicated. After 24 h, the transfected cells were collected and replated into new wells as indicated. Cell proliferation was then determined by CCK-8 assay for the indicated times. All values shown are mean ± SD of triplicate measurements and have been repeated 3 times with similar results (** P < 0.01 at day 4).

To investigate the mechanism by which MYLK-AS1 regulates proliferation and invasion of HCC cells, we tested whether activation of EGFR/HER2-ERK1/2 signaling pathway is responsible for MYLK-AS1 modulation of HCC cell proliferation and invasion. As expected, the EGFR/HER2 inhibitor GW583340 and the MEK1/2 inhibitor PD98059 reduced HepG2 cell proliferation and invasion (Figure 5A and 5B). Importantly, GW583340 and PD98059 abolished the ability of MYLK-AS1 to increase HepG2 cell proliferation and invasion. Moreover, in HepG2 cells, GW583340 and PD98059 decreased phosphorylation of MEK1/2 and ERK1/2, and GW583340 reduced EGFR phosphorylation (Figure 5C), indicating that GW583340 and PD98059 inhibit activation of MEK1/2 and ERK1/2, and GW583340 blocks activation of EGFR. Intriguingly, GW583340 and PD98059 abolished the ability of MYLK-AS1 to stimulate MEK1/2 and ERK1/2. In addition, we used ERK1/2 siRNA and EGFR siRNA to knock down the protein expressions of ERK1/2 and EGFR. Meantime, pcDNA3.0-MYLK-AS1 was used to rescue the inhibitory effect of ERK1/2 and EGFR siRNAs on cell proliferation. The protein expressions of ERK1/2 and EGFR were obviously decreased by their siRNAs (Figure 5D and E). Although cell proliferation was inhibited by knocking down ERK1/2 and EGFR, overexpressing MYLK-AS1 could partially rescue the inhibitory effect (Figure 5 D and E). These results reveal that MYLK-AS1 promotes HCC cell proliferation and invasion through activating the EGFR/HER2-ERK1/2 pathway.

### Knockdown of MYLK-AS1 inhibits tumor growth *in vivo*

**Figure 6:**
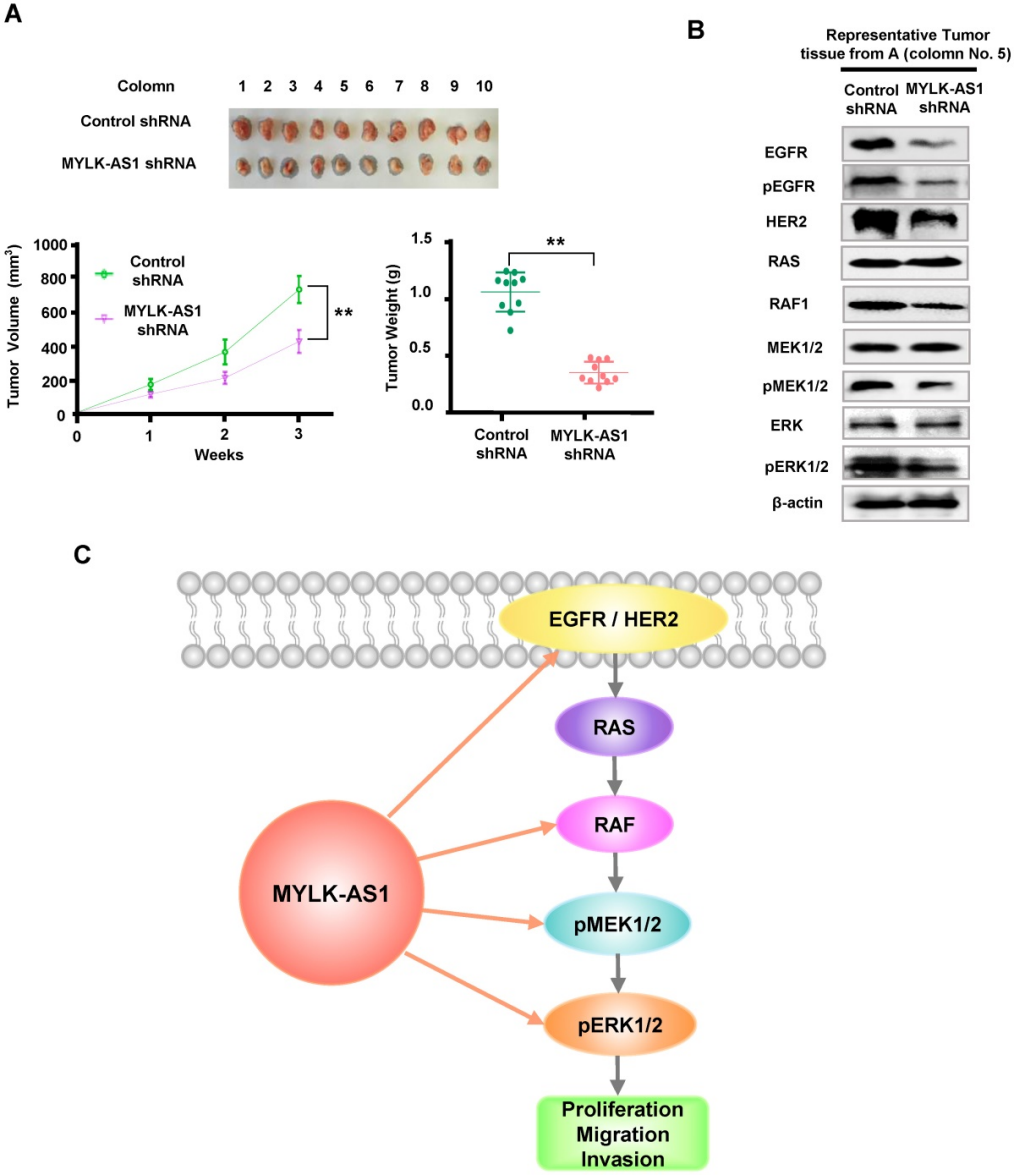
** Knockdown of MYLK-AS1 inhibits tumor growth *in vivo*.** (A) Nude mice were seeded with MYLK-AS1 shRNA-expressing MHCC97-H cells and control shRNA-expressing MHCC97-H cells. The volume of the tumors was calculated every week after transplantation (mean ± SD; n = 10). The mice were killed 21 d after implantation and the weight of the tumors was measured. **P < 0.01 at day 21. (B) Representative immunoblot of the tumors from (A) with the indicated antibodies. (C) A proposed model underlying the role of MYLK-AS1 in hepatoma cell proliferation, migration and invasion via regulation of the EGFR/HER2-ERK1/2 pathway.

For *in vivo* tumor assay, the xenotransplantation model was used to further evaluate the functional effects of MYLK-AS1 on HCC. Compared to the controls, the tumor xenografts produced from MYLK-AS1 shRNA-expressing MHCC97-H cells were significantly inhibited, with smaller tumor volumes and lighter weights (Figure 6A). To further validate the molecular mechanism of MYLK-AS1 in HCC, we detected downstream molecules of EGFR/HER2-ERK signaling pathway of tumor tissues from the xenotransplantation model. As expected, the levels of EGFR, pEGFR, HER2, RAF1, and phosphorylation of MEK1/2 and ERK1/2 were markedly suppressed by downregulating MYLK-AS1 (Figure 6B). Taken together, our results illustrate that knockdown of MYLK-AS1 can inhibit HCC tumor growth *in vivo*.

## Discussion

The EGFR/HER2 signaling pathway is frequently activated in many cancers, including hepatocellular carcinoma, lung cancer, colorectal cancer, and breast cancer, and plays a critical role in carcinogenesis and progression 29-32. Activated EGFR or HER2 predicts poor clinical outcome of cancer patients. In line with their driver roles in cancer development and progression, cancer drugs intercepting EGFR or HER2 currently outnumber therapies targeting other hubs of signal transduction. However, drug resistance is a major obstacle in cancer treatment. Therefore, elucidating the regulation of EGFR and HER2 expression may provide new therapeutic targets for overcoming endocrine resistance or improving clinical outcomes. In this study, we show for the first time that MYLK-AS1 promotes hepatoma cell proliferation, migration and invasion *in vitro*, and tumor formation in nude mice, through upregulation of EGFR and HER2 as well as their downstream targets (Figure 6C). Moreover, MYLK-AS1 was upregulated in liver cancer patients and high MYLK-AS1 expression correlated with poor clinical outcome. Our data suggest that MYLK-AS1 functions as an oncogene and may be a useful molecular target for cancer therapy, especially liver cancer therapy.

The EGFR and HER2 downstream pathways include the RAS-RAF-MEK-ERK pathway, the phosphoinosidyl-3-kinase (PI3K)/protein kinase B (AKT)/mammalian target of rapamycin (mTOR) pathway, the signal transducer and activation of transcription (STAT) pathway, and the phospholipase Cγ (PLCγ)-protein kinase C (PKC) signaling cascade. These pathways control cell proliferation, survival, differentiation, cell adhesion, migration, invasion, metastasis, angiogenesis, and metabolism. Some studies have found that lncRNAs can regulate EGFR/HER2 downstream signaling pathways via microRNAs (miRNAs). For example, lncRNA HOXA-AS3 sponges miR-29c to facilitate cell proliferation, metastasis, and epithelial-mesenchymal transition process, and activates the MEK/ERK signaling pathway in HCC 33. Overexpression of lncRNA H19 attenuates miR-193b-mediated inhibition on multiple driver oncogenes (EGFR, KRAS, PTEN and IGF1R) and MAPK1 gene, and thus triggers EMT and stem cell transformation in HCC 34. Silencing of lncRNA HOXD-AS1 inhibits proliferation, cell cycle progression, migration and invasion of HCC cells through MEK/ERK pathway 35. We show that MYLK-AS1 not only activates MEK/ERK, but also stimulates the expression of both EGFR and HER2, indicating that MYLK-AS1 is a key upstream regulator of EGFR and HER2. It will be interesting to investigate whether MYLK-AS1 regulates EGFR and HER2 expression through miRNAs or other molecules or whether MYLK-AS1 directly regulates the expression of EGFR and HER2. Except for the EGFR/HER2-RAS-RAF-MEK-ERK pathway, whether MYLK-AS1 modulates other above-mentioned pathways also remains to be determined.

RAF kinases are well known oncoproteins that play a key role in cancer development and progression through activation of the MEK/ERK signaling cascade 36. MEK/ERK activation has been shown to be required for mediating the self-renewal capacity and drug-resistant properties of HCC cells, resulting in poor patient survival. Activation of the RAS/RAF/MEK/ERK signaling is present in 50%-100% of HCC tumors 37, yet activating mutations of RAS/RAF are infrequent. The common mechanism by which MEK and ERK are activated by RAF is the downregulation of inhibitory regulators of the pathway in HCC. Our present study showed that MYLK-AS1 dramatically decreases RAF protein expression although the underlying mechanism remains to be investigated.

In summary, our results discover the lncRNA MYLK-AS1 activating EGFR/HER2-ERK signaling pathway and disclose the pivotal role of MYLK-AS1 in promoting HCC cell proliferation, migration and invasion. In the future, exploring the exact roles of MYLK-AS1 in oncogenesis and progression as well as the underlying mechanisms will provide powerful and direct evidence to prove MYLK-AS1 as a therapeutic target for cancer.

## Supplementary Material

Supplementary figure.
