# Supplementary material for: Long noncoding RNA MYLK-AS1 promotes growth and invasion of hepatocellular carcinoma through the EGFR/HER2-ERK1/2 signaling pathway
Source: Int J Biol Sci. 2020 Apr 27;16(11):1989–2000. doi: 10.7150/ijbs.43062 (PMC7211179; doi:10.7150/ijbs.43062)
Supplement: Supplementary file 1 — Supplementary figure. [file ijbsv16p1989s1.pdf]

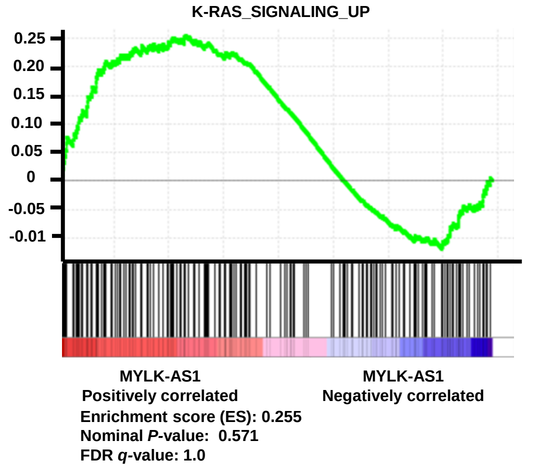

Supplementary Figure 1. GSEA analysis of MYLK-AS1 in HCC adjacent normal tissues. Cluster analysis of MYLK-AS1 did not correlate with the activation of K-RAS signaling in HCC adjacent normal tissues.
